# General Information and Applications of Najuta Fenestrated Stent Grafts for Aortic Arch Aneurysms

**DOI:** 10.3390/jcm14010036

**Published:** 2024-12-25

**Authors:** Seiji Onitsuka, Atsuhisa Tanaka, Hiroyuki Otsuka, Yusuke Shintani, Ryo Kanamoto, Shinya Negoto, Eiki Tayama

**Affiliations:** 1 Department of Surgery, Kurume University School of Medicine, Fukuoka 830-0011, Japan; hiro123@med.kurume-u.ac.jp (H.O.); shintani5212@yahoo.co.jp (Y.S.); kanamotoryo1023@gmail.com (R.K.); negoto_shinya@med.kurume-u.ac.jp (S.N.); eiki@med.kurume-u.ac.jp (E.T.); 2 Department of Thoracic & Cardiovascular Surgery, Saga University School of Medicine, Saga 849-8501, Japan; tanakaatsu1965@gmail.com

**Keywords:** TEVAR, Najuta, fenestrated stent graft, thoracic aortic aneurysm, endovascular aneurysm repair

## Abstract

Endovascular stent graft repair was developed to minimize the invasiveness of open surgery for thoracic and abdominal aortic diseases. This approach involves covering the diseased segment with a stented artificial graft. However, in thoracic endovascular aortic repair (TEVAR) for aortic arch diseases, special consideration is needed to preserve the aortic arch vessels. Standard stent grafts often require additional procedures, such as bypass surgery, to reconstruct the arch vessels. The semi-custom-made Najuta fenestrated stent graft was developed to address this issue. It is a three-dimensional patient-specific stent graft with fenestrations that allow for the preservation of the arch vessels. This study discusses the unique features of the Najuta stent graft and the techniques for its deployment, and it provides an analysis of treatment outcomes based on the current literature.

## 1. Introduction

Surgical treatment for thoracic and abdominal aortic aneurysms and aortic dissection has traditionally relied on graft replacement through thoracotomy and laparotomy. However, conventional open surgery significantly impacts patients physically and mentally due to factors such as surgical incision, bleeding, extended operation time, and the need for cardiopulmonary bypass. Reducing the invasiveness of these surgical procedures is essential in improving the treatment outcomes and patients’ quality of life, as well as in expanding the treatment options for frail patients who are considered high-risk for surgery.

A stent graft for endovascular repair combines a metal stent and a synthetic graft, with its basic structure consisting of a metal stent sewn or bonded to a graft. The stent graft is compacted within a long sheath, which is then inserted into the femoral artery through a small skin incision or percutaneous puncture. Under fluoroscopic guidance, the device is navigated to the lesion site. Once released from the sheath, the stent expands, forcing the graft to adhere closely to the arterial wall, spanning from the proximal to the distal ends of the lesion. This isolates the lesion from the circulating blood, thereby promoting thrombosis. This method confines the blood flow within the stent graft, preventing blood from entering the aneurysm and thus avoiding rupture. In cases of aortic dissection, placing the stent graft in the true lumen seals the intimal tear from the inside, reducing blood flow into the false lumen and preventing its expansion.

In the past quarter century, numerous devices for the endovascular stent graft repair of thoracic aortic aneurysms have been developed and marketed by various companies. These devices have undergone continuous improvements to adapt to diverse pathologies, leading to a steady increase in the number of procedures performed using this method. However, endovascular stent graft repair is not applicable to all thoracic aortic aneurysms. For example, in cases of aortic arch aneurysms where the aneurysm is in close proximity to the aortic arch vessels, it is necessary to preserve the arch vessels while excluding the aneurysm. If the stent graft occludes both the aneurysm and the arch vessels, additional procedures such as bypass surgery are required to restore blood flow to the vessels. Although techniques involving stent grafts with branches to maintain blood flow to the arch vessels have been developed, their widespread use remains challenging.

This article focuses on a Japanese semi-custom-made fenestrated stent graft, which offers a simpler approach to preserving the arch vessels without relying on complex branched techniques, and it provides a detailed description of its structure, application, and advantages.

## 2. History of Stent Grafts

### Introduction of Stent Grafts

The first clinical report on the use of stent grafts was published in 1988 by Volodos et al. from Ukraine. In a Russian academic journal, they described the implantation of a homemade stent graft for a traumatic thoracic aortic aneurysm [1]. In 1991, Parodi et al. from Argentina gained worldwide recognition with their publication in the Annals of Vascular Surgery, detailing the placement of stent grafts composed of a polyester graft and balloon-expandable metal stents in five cases of abdominal aortic aneurysms [2]. Subsequently, from 1994 to 1996, Dake et al. reported stent graft treatment for thoracic aortic aneurysms, contributing to the gradual global adoption of this treatment method [3,4,5].

## 3. Development of Stent Grafts in Japan

**Figure 1:**
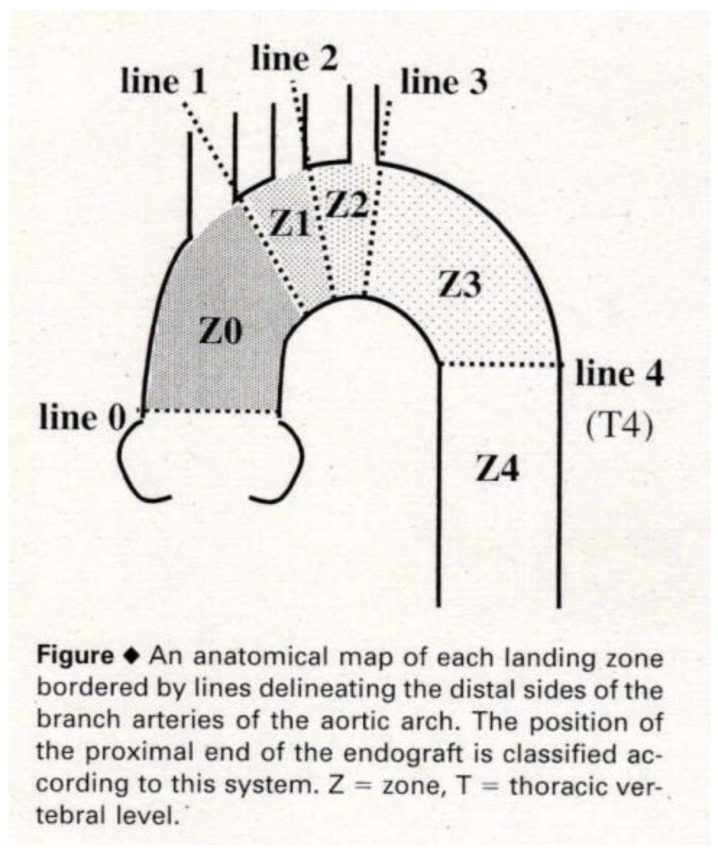
The roundtable discussion of the First International Summit TOWSES 2001 established anatomical zone mapping for the aortic arch.

In Japan, Ishimaru and colleagues began developing stent grafts in 1995, manufacturing a self-expanding stent graft using stainless-steel Z-stents and polyester grafts and achieving notable treatment outcomes for thoracic aortic aneurysms [6]. Concurrently, Kato et al. reported the effectiveness of their homemade stent grafts in reducing the false lumen size by closing the primary entry in aortic dissections [7]. Inoue et al. also detailed their branched stent grafts for aortic arch diseases [8]. Inspired by these pioneering efforts, we also initiated this treatment in 1999 under the mentorship of Tokyo Medical University [9,10]. In 2001, Ishimaru organized the First International Summit TOWSES 2001 in Tokyo, inviting international leaders in stent graft technology. The roundtable discussion there led to the establishment of anatomical zone mapping for the aortic arch, which remains in use today (Figure 1) [11].

Internationally, the terminology for endovascular stent graft repair once varied, with terms such as the “transluminal placement of endovascular stent grafts (TPEG)” being used. However, the “Reporting Standards for Endovascular Aortic Aneurysm Repair” published in 2002 [12] and the subsequent U.S. guidelines published in 2003 [13] standardized the terminology for abdominal stent graft insertion as “endovascular aneurysm repair (EVAR)”. Later, thoracic stent graft insertion was termed “thoracic endovascular aortic repair (TEVAR) [14]”, reflecting the broader range of thoracic conditions that it addresses, including true aneurysms, dissections, trauma, and esophageal cancer infiltration.

## 4. Approval of Commercial Devices in Japan

**Figure 2:**
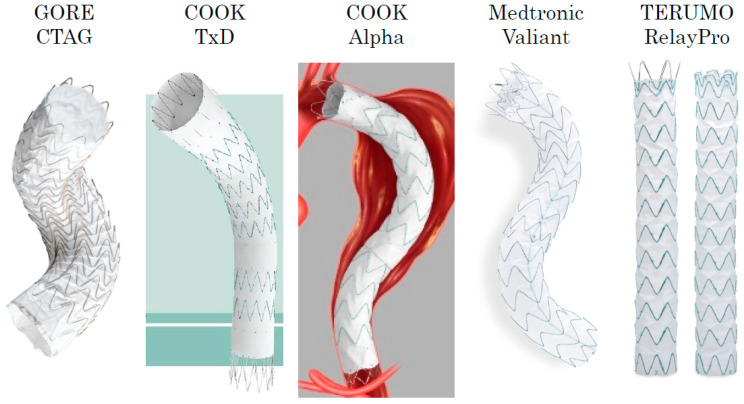
The CTAG (W. L. Gore & Associates, Inc., Flagstaff, AZ, USA) device is a stent graft composed of an expanded polytetrafluoroethylene (ePTFE) tube reinforced with ePTFE/fluorinated ethylene propylene film and supported by a self-expanding nitinol wire frame along its external surface (image courtesy of W. L. Gore & Associates G. K.). The Zenith dissection endovascular system (TxD) (Cook Inc., Bloomington, IN, USA) is a dedicated design for type B aortic dissection. This design is a composite design using a proximal stent graft and bare metal stent. The proximal stent graft is constructed by a knitted polyester graft and a stainless-steel-based stent. The bare metal stent is constructed with a stainless-steel Z-stent. This bare stent promotes immediate true lumen expansion and aortic remodeling (image courtesy of Cook Inc.). The Alpha thoracic stent graft (Cook Inc., Bloomington, IN, USA) was constructed by covering a self-expanding nitinol Z-stent with a thin knitted polyester graft. The Alpha has proximal and distal components with bare alignment stents (image courtesy of Cook Inc.). The Valiant stent graft system (Medtronic Inc., Minneapolis, MN, USA) is a modular, self-expanding, tubular endoprosthesis. The nitinol scaffolding of the stent graft is composed of a series of serpentine five-peaked springs stacked in a tubular configuration. The scaffolding in this device is sewn to the outside of the graft material (image courtesy of Medtronic Inc.). The RelayPro (Terumo Aortic; formerly Bolton Medical, Sunrise, FL, USA) device is composed of self-expanding electro-polished nitinol, sinusoidal stents that are sutured to tightly woven polyester graft fabric for profile reduction. It has two types of devices, which have a bare stent and a non-bare stent (NBS) proximal configuration (image courtesy of Terumo Corporation).

In 2006, the Zenith stent graft (Cook Inc., Bloomington, IN, USA) was the first EVAR device to receive domestic approval in Japan. Subsequently, the Gore TAG stent graft (W. L. Gore & Associates, Flagstaff, AZ, USA) was approved in 2008 as the first commercially available TEVAR device. This first approval occurred 10 years after the approval in Europe and 3 years after that in the United States. Since then, multiple commercial TEVAR devices have been approved in Japan, and currently, more than five different devices are available for clinical use. The thoracic endovascular stent grafts currently on the market consist of self-expandable Z-shaped metal stents made of nitinol or stainless steel and grafts made of woven polyester or polytetrafluoroethylene (PTFE) material (Figure 2). The stent grafts for TEVAR approved in Japan are summarized in Table 1.

With these developments, the homemade stent grafts that had been used in Japan for over a decade were phased out. However, the team from Tokyo Medical University, led by Ishimaru, Kawaguchi, and Yokoi, sought to preserve and further enhance the concept of their homemade fenestrated stent grafts, which had demonstrated highly favorable results in treating various aortic arch diseases. Their efforts led to the development of the Najuta stent graft (SB-Kawasumi Laboratories, Inc., Kawasaki, Japan) [15,16,17,18].

## 5. Thoracic Endovascular Aortic Repair (TEVAR)

### 5.1. Stent Graft Eligibility Criteria

The proportion of TEVAR use in the surgical treatment of aortic diseases is increasing annually. However, stent grafts are not suitable for all aortic diseases. To effectively seal and isolate the aortic lesion, the stent graft requires adequate regions of the arterial wall to attach securely to both the proximal and distal sides. These regions, referred to as “landing zones”, must generally have a length of at least 20 mm, with a favorable morphology and arterial wall characteristics.

In TEVAR, the proximal landing zone extends from the aortic arch vessels (such as the brachiocephalic artery, left common carotid artery, or left subclavian artery) to the proximal end of the aortic disease. The distal landing zone extends from the distal end of the aortic disease to the major abdominal vessels (such as the celiac artery or superior mesenteric artery) or the Adamkiewicz artery. If these proximal and distal landing zones are not adequately secured, the stent graft may not adhere properly to the arterial wall, leading to “endoleaks” in which blood leaks into the treated area. Uncontrolled endoleaks can result in serious complications, such as rupture in cases of aortic aneurysms. Therefore, each device has specific anatomical requirements, known as instructions for use (IFUs), to ensure proper application.

### 5.2. TEVAR for Aortic Arch Aneurysm

In many cases, TEVAR for distal aortic arch aneurysms can secure the proximal landing zone up to Zone 2 by deploying a stent graft just distal to the left common carotid artery (LCA) (Figure 1). The left subclavian artery (LSA) ostium is covered with a stent graft along with the aneurysm, and the proximal portion of the LSA is occluded with metallic coils to prevent retrograde flow into the aneurysm (type II endoleak). Preoperative evaluations of the intracranial artery, carotid artery, and vertebral artery are performed in cases covering the LSA, with consideration given to LSA revascularization.

When the aneurysm treatment cannot secure a proximal landing zone up to Zone 2, a two-debranch TEVAR is performed by deploying a stent graft just distal to the brachiocephalic artery (BCA) (Zone 1) (Figure 1), thereby preserving the blood flow to the BCA while closing the LCA and LSA. Additionally, a bypass from the right axillary artery to the left axillary artery and the LCA is conducted [19].

For aneurysms extending further proximally, procedures such as total-debranch TEVAR are performed under a median sternotomy with three bypass grafts from the ascending aorta to the BCA, LCA, and LSA, followed by the placement of a stent graft up to Zone 0 of the ascending aorta (Figure 1) [19]. Alternatively, the chimney technique is utilized, where a small covered stent is placed alongside the main stent graft from the BCA while performing a two-debranch bypass [20]. This combined approach of TEVAR and bypass surgery for aortic vessels is referred to as hybrid TEVAR. The hybrid TEVAR, performed in cases where the distance from the arch vessels to the aneurysm is short and a proximal landing zone cannot be secured, is more surgically invasive than standard TEVAR. Concerns remain regarding the patency rate of anti-anatomical bypass grafts, and there is an increased risk of stroke during the procedure. Consequently, there has been growing interest in the development of stent grafts with fenestrations or side branches, which preserve blood flow to the arch vessels while excluding the aneurysm.

## 6. TEVAR Using Semi-Custom-Made Najuta Fenestrated Stent Graft for Aortic Arch Aneurysm

### 6.1. Development History and Commercialization of Fenestrated Stent Grafts

**Figure 3:**
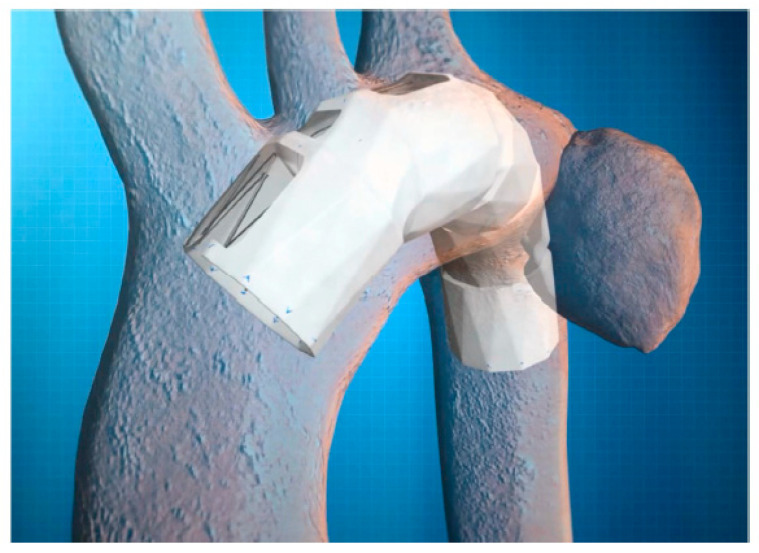
This image depicts TEVAR using the semi-custom-made Najuta fenestrated stent graft for the aortic arch aneurysm. The Najuta extends the proximal landing zone into the ascending aorta, enabling the exclusion of the aortic arch aneurysm while preserving the arch vessels through fenestrations. TEVAR: thoracic endovascular aortic repair.

The team at Tokyo Medical University began crafting homemade stent grafts in 1995, initially without fenestrations. They developed stent grafts by sewing an ultrathin woven polyester graft (Ube Industries, Tokyo, Japan) onto a self-expanding stainless-steel stent (Gianturco Z stent; Cook Medical, Bloomington, IN, USA). Initially, the focus was on flexibility to adapt to the shape of the aorta in individual patients. However, although these flexible stent grafts were firmly fixed in the early postoperative period, migration due to blood flow was observed during follow-up [10,15]. Histopathological studies confirmed that a perigraft space with loosely arranged blood cells persisted, and no histological support for the firm biological fixation of endografts was provided even years after implantation [21]. Based on these experiences, they improved the stent graft to conform three-dimensionally to the curvature of the aortic arch. Furthermore, they extended the proximal landing zone into the ascending aorta to prevent not only migration but also type Ia endoleaks by creating fenestrations to preserve the arch vessels (Figure 3).

Building on the concept of homemade stent grafts developed by the Tokyo Medical University team, Kawasumi Laboratories successfully manufactured a fenestrated stent graft as an industrial product. Clinical trials began in 2008 across 11 institutions involving 117 cases. By 2012, the stent graft had received approval and was launched on the market. The name “Najuta” is derived from “Nayuta”, a unit of measurement in East Asian culture representing an extremely large number (10^60^ or 10^72^) almost approaching infinity.

### 6.2. Structure of Najuta Stent Graft

**Figure 4:**
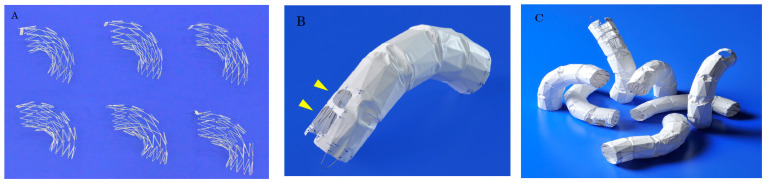
(**A**), The Najuta fenestrated stent graft is a three-dimensional, patient-specific device designed with fenestrations to preserve the aortic arch vessels. The stainless-steel stent framework is pre-shaped to conform to the aortic morphology. (**B**), The PTFE graft is attached to the exterior of the stents solely at the proximal and distal regions of the stents and around the fenestrations (arrows). Up to three fenestrations can be created, and they can be adjusted anteriorly and posteriorly to align with the arch vessels. (**C**), The Najuta offers approximately 760,000 specifications, allowing it to conform similarly to mold to the aortic morphology in most patients while aligning the fenestrations with the arch vessels. PTFE: polytetrafluoroethylene.

The Najuta stent graft is constructed by covering a self-expanding stainless-steel Z-stent with a PTFE graft. The 25 mm long Z-stent is assembled by joining two struts on the greater curvature side and connecting four to five sections together, thereby creating stent frameworks of various shapes (Figure 4A). After confirming the aortic shape using 3D-CT, the most suitable stent framework is selected and covered with a PTFE graft.

Based on extensive experience with aortic shapes, 68 types of stent structures have been prepared, with graft diameters ranging from 24 mm to 46 mm in both straight and tapered types (−4 mm and −6 mm). The maximum length of a stent graft is approximately 18 cm. The fenestrations for the preservation of arch vessels range in size from approximately 12 mm to 18 mm squared, with one to three fenestrations available (Figure 4B). These fenestrations can be adjusted to the patient’s arch vessels by shifting them anteriorly or posteriorly by 2 mm each up to a maximum of 14 mm. Additionally, distal types without fenestrations are available for descending aortic diseases. With these combinations, there are, theoretically, approximately 760,000 different specifications, allowing the stent to conform to the shape of most patients’ aortas while aligning the fenestrations to the arch vessels (Figure 4C).

### 6.3. Fabrication of Najuta

First, axial cross-sectional image data from contrast-enhanced CT scans taken in slices of 2 mm or less are provided to SB Kawasumi. SB Kawasumi analyzes these CT data to construct 3D images, confirming the morphologies of the proximal and distal landing zones, as well as the size and position of the fenestrations that align with the arch vessels. The IFUs specify that the length of the proximal and distal landing zones should be at least 20 mm, with an inner diameter between 20 mm and 40 mm, and that the access vessels for sheath insertion should not exhibit significant calcification or severe tortuosity. These criteria are carefully considered in consultation with the supervising physician.

**Figure 5:**
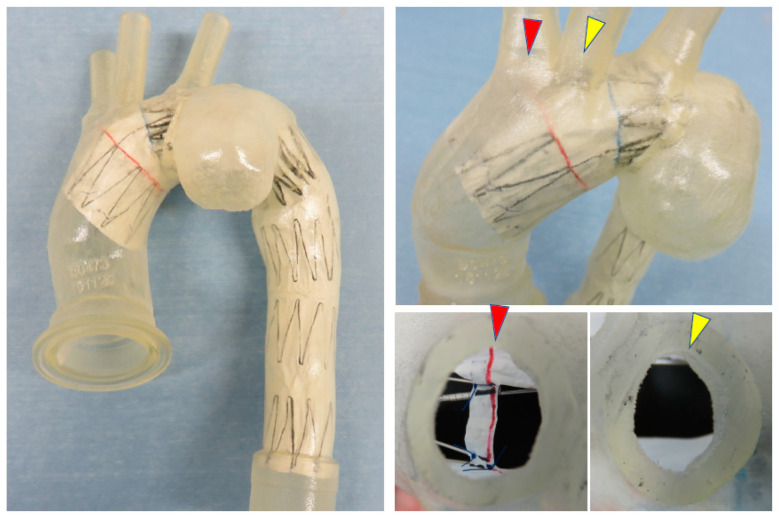
Using CT data, a 3D-printed vascular model of a patient is fabricated, allowing for the placement of the prototype Najuta to assess its conformance and alignment with the fenestrations for the brachiocephalic artery (red arrows) and the left common carotid artery (yellow arrows).

**Figure 6:**
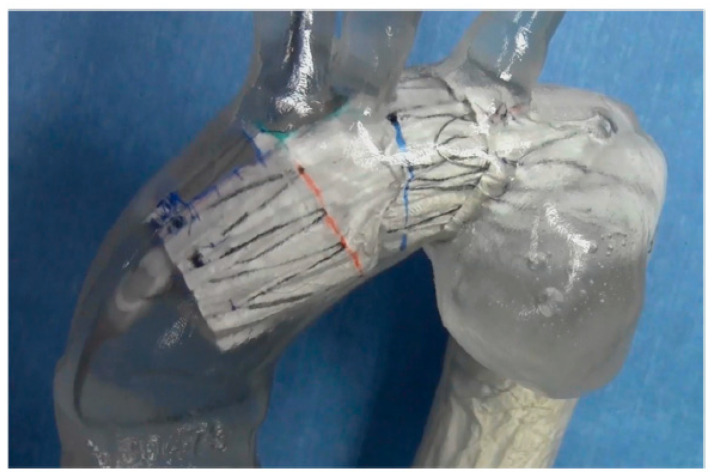
The surgeon can test the deployment technique for the prototype Najuta under a pulsatile water flow within a 3D-printed vascular model in SB-Kawasumi Laboratories, Inc. (Kawasaki, Japan) prior to surgery.

If suitable, a recommended deployment plan is created, detailing the diameter and shape of the Najuta stent graft, as well as the size and position of the fenestrations. Additionally, a 3D-printed vascular model of the patient is created from the CT data, allowing for the placement of a prototype Najuta to assess its conformity and the alignment of the fenestrations (Figure 5). SB Kawasumi also has the capability to test the deployment technique of the prototype Najuta under a pulsatile water flow within a 3D-printed vascular model (Figure 6).

Since the Najuta is semi-customized for each patient, it takes approximately four weeks from the submission of the CT data to complete the device.

### 6.4. The Effect of Extending the Proximal Landing Zone with Najuta

**Figure 7:**
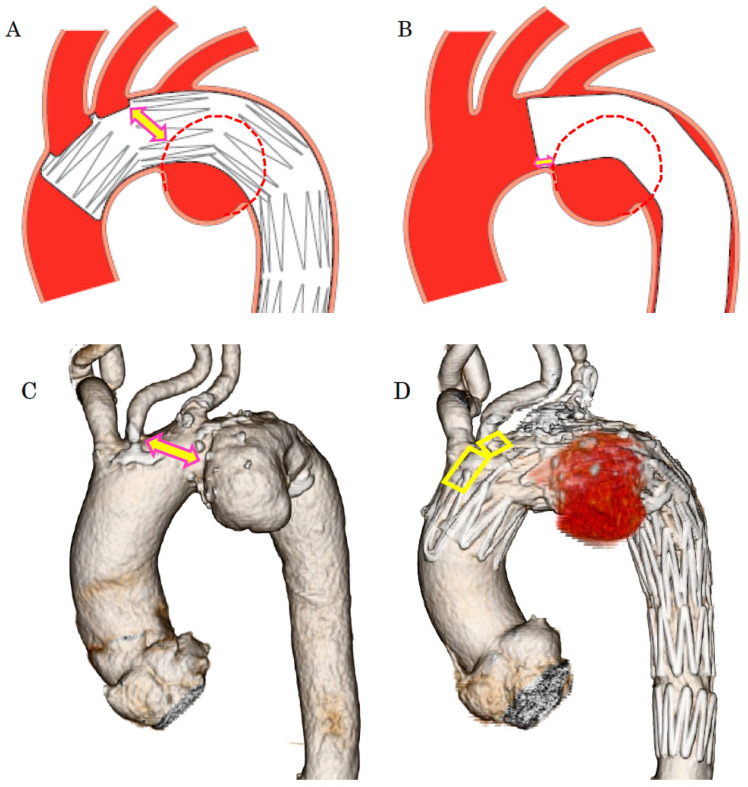
When applying the Najuta stent graft to an aortic arch aneurysm, its proximal end can extend into the ascending aorta, preserving both the brachiocephalic artery and the left common carotid artery through two fenestrations (squares) (**D**). Consequently, the distance from the left common carotid artery to the aneurysm serves as the proximal landing zone (sealing zone) (double-ended arrows), which is typically longer (**A**,**C**) than that with conventional stent grafts (**B**).

When performing TEVAR for an aortic arch aneurysm while preserving the BCA and the LCA, the distance from the LCA to the aneurysm constitutes the proximal landing zone. In conventional Zone 2 TEVAR using a standard stent graft, the shortest distance from the proximal edge of the stent graft to the lesion defines the proximal landing zone. In contrast, with the Najuta, the stent graft extends into the ascending aorta while preserving the BCA and LCA through two fenestrations. Thus, the distance from the fenestration for the LCA to the aneurysm serves as the proximal landing zone (sealing zone). For aneurysms located on the lesser curvature of the aortic arch away from the LCA, the proximal landing zone can be extended compared to a standard stent graft (Figure 7A–D). This suggests that the Najuta has the potential to expand the indications for TEVAR to include more proximally located aortic arch aneurysms and increase the likelihood of avoiding debranching bypass surgery.

Although it is an off-label use, there are many aortic arch diseases in which the Najuta is effective. For pseudoaneurysms at the anastomotic site following ascending aorta or aortic arch replacement surgery, TEVAR with a standard stent graft may be challenging; however, the Najuta can sometimes be placed across the anastomosis while preserving the arch vessels with fenestrations [22,23]. Furthermore, in TEVAR for type B aortic dissection, the Najuta can leverage its features of a low-radial-force inner stent skeleton and custom design, which accommodates the steep distal aortic arch and the narrow deformed true lumen [24,25]. Additionally, it is highly anticipated to be applicable for blunt aortic injuries, which commonly occur at the aortic isthmus, and for conditions involving the right aortic arch [26,27].

### 6.5. Stability and Patency of Fenestrations with Placement in the Ascending Aorta

In TEVAR for aortic arch aneurysms, the Najuta stent graft achieves superior positional stability without the need for a proximal bare stent or other mechanical attachment systems due to its extension into the ascending aorta and its three-dimensionally shaped stent skeleton. This structure reduces migration between the intraoperative and long-term postoperative periods, maintaining the patency of the fenestrations. Clinical trial cases have utilized preoperatively fabricated 3D vascular models and compared them with post-implantation CT scans to confirm the alignment rate of the arch vessels and fenestrations. According to SB Kawasumi’s post-marketing surveillance (PMS) conducted from its market release in 2013 to 2022, there have been no cases of occlusion at the BCA fenestration, with only a few early cases of occlusion at the LCA fenestration. As of 2016, the fenestrations can be adjusted up to 14 mm anteriorly and posteriorly to align more precisely with the patient’s arch vessels, significantly enhancing the accuracy of fenestration alignment with the arch vessels.

### 6.6. Suppression of Type I Endoleak via “Passive Sealing”

The PTFE graft covering the Najuta’s stent skeleton is intentionally sutured to the stent only at the proximal and distal ends, as well as around the fenestrations (Figure 4B). This design allows the soft graft material to be stretched by the blood flow, adhering closely to the aortic wall in resemblance to a sail—an effect known as “passive sealing”. This close sealing is particularly expected to suppress type I endoleaks when the proximal and distal landing zones are reverse-tapered or of poor quality [28].

### 6.7. The Method of Deploying the Najuta

Due to the unique characteristics of the device, the deployment technique for the Najuta requires careful attention to several key aspects that differ from standard stent grafts.

**Figure 8:**
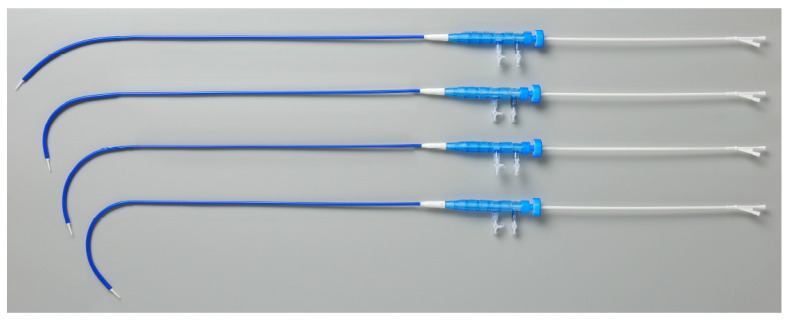
The low-profile sheath houses the Najuta within its proximal portion. The proximal portion of the sheath has a curved shape with four curvatures to match the pre-shaped stent grafts.

Sheath Shape: The delivery system houses the Najuta, which is custom-made to match the shape of the aorta, in the proximal part of the sheath. Four curved sheath shapes are prepared. The outer diameter of the proximal housing part varies with the stent graft diameter: 21 Fr for diameters of 24–30 mm, 22 Fr for diameters of 28–34 mm, 23 Fr for diameters of 32–42 mm, 24 Fr for diameters of 40–42 mm, and 25 Fr for diameters of 44–46 mm. The distal shaft part is thinner, with an outer diameter of 18 Fr, making it a low-profile device compared to other standard devices (Figure 8). Due to the diameter difference between the proximal and distal parts of the sheath and the soft proximal tip, percutaneous femoral artery puncture insertion is challenging. Generally, the femoral artery (or external iliac artery) is accessed via skin incision, and both the proximal and distal parts are tightened for hemostasis with tourniquets. The sheath is inserted through a transverse incision in the femoral artery. When the proximal 21–25 Fr part of the sheath is inserted and reaches the 18 Fr shaft part, bleeding may occur from the insertion site. At this point, hemostasis is achieved by tourniqueting the femoral artery proximally and distally.

Percutaneous Insertion: When performing percutaneous insertion, it is possible to insert the GORE DrySeal Flex Introducer Sheath (W. L. Gore & Assoc., Flagstaff, AZ, USA) percutaneously and then insert the Najuta delivery system through this sheath. Since the proximal diameter of the Najuta, which is placed up to the ascending aorta, is usually 36 mm or larger, the 24 Fr GORE DrySeal sheath is compatible with the 23 Fr Najuta sheath. Therefore, the femoral and external iliac arteries must have a diameter and condition that allow the passage of the 24 Fr GORE DrySeal sheath, which has an outer diameter of 8.8 mm. There have been instances where insistence on percutaneous insertion has led to access route injuries.

If the iliac arteries are not suitable for the passage of the Najuta sheath alone, it is more reliable to expose the infrarenal abdominal aorta through a laparotomy, perform double tobacco-pouch sutures on the anterior aortic wall, and then insert the GORE DrySeal sheath as a conduit for the insertion of the Najuta sheath. Using the same technique from the common iliac artery may result in arterial stenosis after the tobacco-pouch sutures.

**Figure 9:**
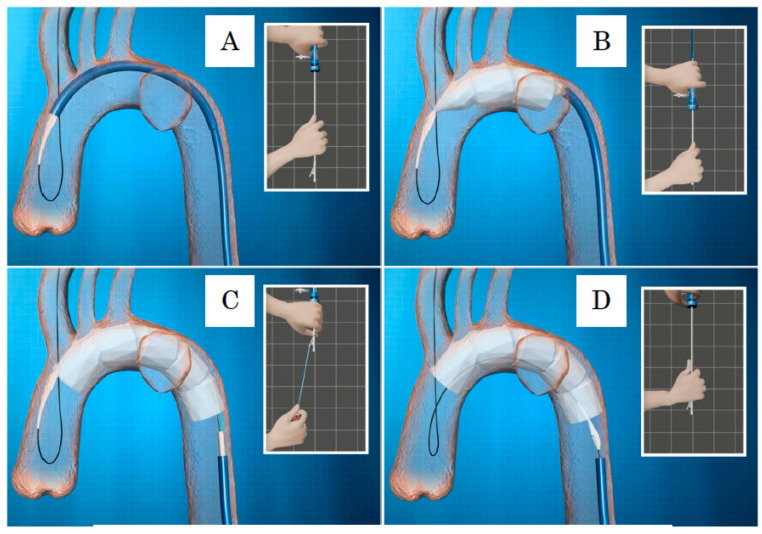
(A) When the proximal tip reaches the ostium of the brachiocephalic artery, the sheath is advanced further as the guidewire is slackened into the ascending aorta. (**B**) While holding the inner rod of the delivery system, manually pulling down the outer sheath allows the Najuta to be deployed from the proximal side through the expanding force of the stent. (**C**) After completing the deployment to the distal end, one must pull the stabilizer line, which constricts the proximal end, to fully release the proximal end of the Najuta. (**D**) Subsequently, by pulling the inner rod caudally, the U-shaped hook is disengaged from the proximal tip, completing the placement.

Tug-of-wire method: When inserting the Najuta sheath into the aorta, it is necessary to straighten the curved sheath by pulling both proximally and distally on a long guidewire (0.032 inches in diameter, 400 cm long) that passes through the femoral artery to the right brachial artery. This technique is known as the “tug-of-wire method” [6]. By adjusting the tension of the pull-through guidewire, the sheath is inserted, allowing it to rotate naturally according to the shape of the aorta. When the proximal tip reaches the ostium of the BCA, the sheath is advanced further as the guidewire is slackened into the ascending aorta (Figure 9A).

A 6 Fr twin-port sheath is placed in the right brachial artery, utilizing the main port for the tug-of-wire route, and a 4 Fr pigtail catheter is inserted through the side port and placed in the ascending aorta for angiography. Since the LSA is covered in many cases, the arterial pressure line is secured in the right radial artery, although the arterial pressure can also be measured from the flush port of the twin-port sheath.

In some cases, such as those with prior ascending aorta graft replacement surgery, advancing the Najuta sheath into the ascending aorta can be challenging. In such situations, the right brachial artery sheath can be replaced with a long guiding catheter (7–8 Fr in diameter, over 70 cm long), which is advanced to the ascending aorta while pushing the pull-through guidewire proximally and advancing the Najuta sheath [22,29]. If this remains difficult, the guidewire used in the tug-of-wire method can be exchanged for a stiff guidewire, with its proximal end grasped by a snare catheter inserted from the contralateral femoral artery and pulled caudally to advance the sheath using the “Lasso method” [30]. Another technique involves inflating an aortic balloon inserted from the contralateral femoral artery on the greater curvature side of the aortic arch, pressing the Najuta sheath against the lesser curvature while advancing it [29].

**Figure 10:**
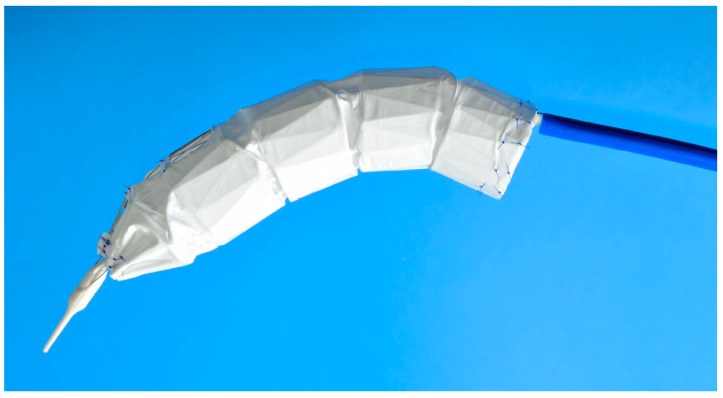
The U-shaped hook mounted at the proximal end of the Najuta is secured within the tip of the inner rod, and the proximal end of the Najuta is constrained by the stabilizer line, making its position easily adjustable during placement.

Deployment of the Najuta: While holding the inner rod of the delivery system, manually pulling down the outer sheath allows the Najuta to be deployed from the proximal side through the expanding force of the stent. When two to three stents are deployed, the device is pushed down by the blood flow (Figure 9B). To account for this tendency, deployment should begin slightly proximal to the intended target zone. Even if the device is pushed down by the blood flow, the U-shaped hook attached to the proximal end of the stent remains secured in the groove of the proximal tip. Additionally, the proximal end of the Najuta is constricted by the stabilizer line during deployment, which facilitates the easy adjustment of the placement position (Figure 10). By counteracting the push from the blood flow, the greater curvature side of the Najuta adheres more closely to the greater curvature of the aortic arch, and the proximal part tilts toward the lesser curvature, reducing the bird-beak effect. Furthermore, during deployment, the PTFE graft acts like a sail under the influence of the blood flow, enhancing the attachment to the arterial wall [28].

**Figure 11:**
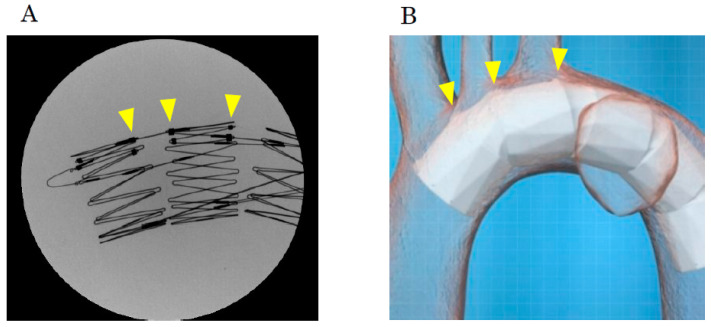
(**A**) Under radiographic fluoroscopy, the position of the fenestrations can be confirmed using multiple markers where stent struts are connected on the greater curvature side (arrows). (**B**) When the stent markers are aligned with the arch vessels according to the placement plan, the fenestrations naturally align with the arch vessels (arrows).

Although temporary rapid pacing can be used to prevent the device from being pushed down by the blood flow during deployment, it is important to adhere to the above principles. Under fluoroscopy, the stent skeleton is clearly visible, and the positions of the fenestration markers, which connect the stent frames on the greater curvature side, can be confirmed [24] (Figure 11A). By marking or creating a roadmap on the angiography image and aligning the fenestration marker with the ostia of the arch vessels according to the preoperative plan, the fenestrations can be automatically aligned with the arch vessels (Figure 11B). The three-dimensional stent structure ensures that as long as the fenestration marker is aligned with the arch vessels in the longitudinal direction, there is no misalignment due to anterior–posterior rotation.

After completing the deployment to the distal end, one must pull the stabilizer line, which constricts the proximal end, to fully release the proximal end of the Najuta (Figure 9C). Subsequently, by pulling the inner rod caudally, the U-shaped hook is disengaged from the proximal tip, completing the placement (Figure 9D).

## 7. Post-Deployment Procedure

Sheath retrieval and post-placement angiography: Due to the Z-shaped stent skeleton inside the Najuta, it is important to exercise caution when retrieving the inner rod after placement. The proximal tip attached to the inner shaft can easily catch on the stent skeleton, so the inner rod should be gently pulled down while rotating it to change the orientation of the proximal tip. Special care is needed when navigating through the lesser curvature of the distal arch and storing it within the outer sheath. To prevent accidental strong pulling in cases of sticking, the proximal tip is designed to detach intentionally from the inner shaft. As long as the tug-of-wire technique is maintained, the detached proximal tip remains on the pull-through guidewire, allowing for retrieval with a long catheter inserted from the right brachial artery and pushed through to the femoral artery. If the wire is released and the proximal tip falls off, a snare catheter should not be opened inside the Najuta to avoid entanglement with the stent skeleton, which can complicate removal.

The process of removing the inner rod should be conducted under fluoroscopic observation, ensuring that the pull-through guidewire is kept bent in the ascending aorta. The pull-through guidewire, after placement, passes through the Najuta lumen, exits at the proximal end, and reaches the BCA (Figure 9D). Pulling the guidewire in this state will result in the caudal migration of the Najuta. The 4 Fr pigtail catheter, raised into the BCA during placement, should be positioned in the ascending aorta via the fenestration of the BCA, guided by the wire, for final angiography.

**Figure 12:**
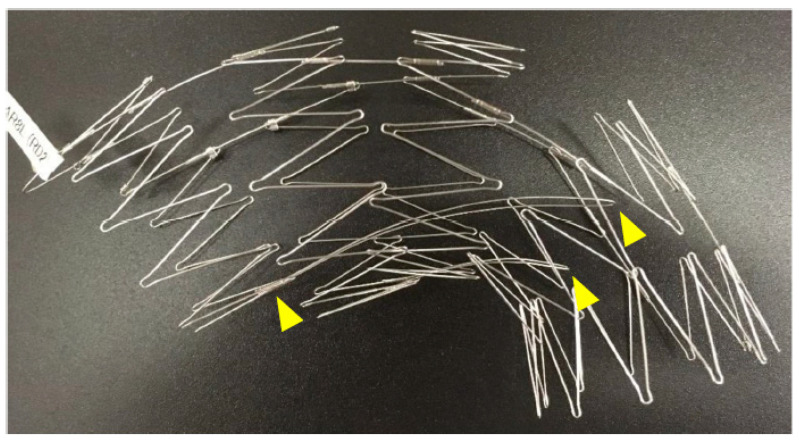
The fin skeleton (usually with two fins) (yellow arrows) used to stabilize the Najuta during deployment, running from the lesser curvature of the first stent to the fourth stent.

Touch-up balloon: A touch-up balloon is used if a type Ia endoleak is observed on the angiography or if there is poor expansion of the stent skeleton. When performing a touch-up balloon, it is crucial to be aware of the stent and the fin skeleton (which usually has two fins) running from the lesser curvature of the first stent to the fourth stent (Figure 12). The fins stabilize the Najuta during deployment, but ballooning in the part of the fourth stent can push these fins against the lesser curvature, causing the proximal portion to extend toward the greater curvature. This behavior can exacerbate the bird-beak effect at the proximal portion. The collapse of the first stent due to blood flow has been reported [31], possibly due to the size of the bird beak.

Without a mechanical attachment system, the Najuta is prone to migration during balloon inflation. The use of the GORE Tri-Lobe Balloon Catheter (W. L. Gore & Assoc., Flagstaff, AZ, USA), which is less prone to being pushed by blood flow, is recommended. Touch-up ballooning should never be performed under the pull-through guidewire; one must always switch to a stiff guidewire and carefully apply proximal pressure with the balloon.

Placement of distal stent grafts and other endovascular treatments: After placing the Najuta, additional stent graft placement may be required intraoperatively or postoperatively due to insufficient distal landing zones or new lesions of the descending aorta. If additional distal stent graft placement is anticipated during surgery, it is common to prepare an additional standard stent graft of the same or one-step-larger diameter than the distal part of the Najuta. As with touch-up balloons, attention must be paid to the presence of fins during additional stent graft placement.

In cases where the treatment length is long and a single Najuta is insufficient for the distal landing zone, a distal stent graft is first placed, followed by the fenestrated Najuta. The distal stent graft should preferably be a device without a proximal bare stent so as to avoid disrupting the sealing of the proximal Najuta.

Many patients requiring TEVAR for an aortic disease also require endovascular treatment for coronary artery disease, valvular disease, carotid arteries, or cerebral arteries. It is essential to recognize that these procedures may become challenging or risky after the placement of the Najuta.

Type Ia endoleak: If a type Ia endoleak is detected on post-placement angiography, a touch-up balloon is considered first. Minor endoleaks often resolve themselves naturally on postoperative CT, and additional stent graft placement should be carefully considered, weighing the risks. A persistent type Ia endoleak in cases with a proximal landing zone of less than 20 mm is often due to an endoleak from a fenestration near the lesion. In cases where the endoleak site is difficult to identify on postoperative CT, further evaluation using 4D-CT or Angio-CT may be considered. If a type Ia endoleak is confirmed via direct angiography, embolization with metallic coils can be performed between the stent graft and the arterial wall, although this technique can be technically challenging and potentially ineffective.

When placing an additional stent graft for a type Ia endoleak, one should use a stent graft with a proximal bare stent, positioning the graft just below the fenestration. This allows the bare stent to better align the fenestration.

Conversion to open surgery: There have been instances requiring conversion to open surgery after Najuta placement due to type Ia endoleaks, aneurysm expansion, or type A aortic dissection. CT should confirm the position of the first and second stents of the Najuta, placed from the ascending to the proximal arch, ensuring readiness for procedures involving ascending aorta clamping or cardiopulmonary bypass perfusion routes. If the Najuta itself demonstrates no defects or infections, partial replacement can be performed by resecting the proximal portion. To navigate the distal anastomosis with the Najuta, the PTFE graft is cut cross-sectionally between the stents under open distal conditions, and the struts connecting the stents are severed with wire cutters. The fins can be removed along with the stents.

## 8. Complications of Najuta

Cerebral infarction: Similar to the complications seen in open surgery for thoracic aortic diseases, TEVAR can have severe and potentially fatal complications. One major concern is cerebral infarction or organ ischemia caused by the detachment of the atheroma or thrombus during the insertion of the delivery system or the placement of the stent graft in the aortic arch. In particular, in cases of a shaggy aorta, gentle manipulation is crucial, and careful consideration of the surgical indications is required [32].

PMS results indicate that the incidence of cerebral infarction is 4–5%, with no reported cases of cerebral infarction due to fenestration misalignment during the follow-up period.

Intimal injury (stent graft-induced new entry: SINE) and retrograde type A aortic dissection (RTAD): A new intimal tear caused by the placement of a stent graft is known as a stent graft-induced new entry (SINE). This can lead to dissection in a true aneurysm or create new blood flow into the false lumen in an aortic dissection. A SINE is more likely to occur when a stent graft with insufficient flexibility is placed in a curved portion or when a stent graft with an excessively oversized diameter relative to the aorta is used. SINEs can occur at both the distal and proximal ends. If a SINE occurs at the proximal end and the dissection extends to the ascending aorta, it can result in a life-threatening condition known as retrograde type A aortic dissection (RTAD), which requires emergency surgery [32].

Intimal injury can also occur due to aortic clamping in open surgery. In TEVAR, it is crucial to have a thorough understanding of the arterial characteristics and morphology and to select the appropriate device and placement plan. Monitoring with CT or other imaging modalities is necessary during the procedure and over the long-term postoperative period. Intimal injury can also occur during delivery system manipulation, especially in TEVAR for aortic dissection cases, where meticulous care is required when handling the sheath within the aortic arch.

According to PMS results, the incidence of RTAD and distal SINE is less than 1%. The Najuta’s inner stent has a low radial force, which may reduce the risk of intimal injury. However, since the length of one Z-stent is 25 mm, its flexibility is inferior to that of standard stent grafts with shorter stent lengths.

## 9. Clinical Outcomes of Najuta (Table 2)

Kawaguchi et al. reported on the treatment outcomes of 682 patients who underwent TEVAR using homemade stent grafts prior to the commercial availability of Najuta in 2008. The stent grafts were progressively improved over time, with fenestrated stent grafts, which later became the prototypes for the Najuta, used in 288 patients. Across all 682 patients, the initial success rate was 95.2%, the incidence of cerebral infarctions was 3.8%, and no cases involving a fenestrated stent graft resulted in the occlusion of the arch vessel. Among the patients observed for more than two years, 62% exhibited aneurysm reduction and 5% showed enlargement. The stent graft-related complication rate was 8.4%, with a device fracture rate of 1.4% and a migration rate of 7% [15].

**Table 2:** Literature overview of Najuta fenestrated and TBE branched TEVAR in the aortic arch. * Total 682 homemade stent grafts (288 of which were treated with fenestrated stent grafts). ^†^ Initial success rate. RTAD: retrograde type A aortic dissection.

Device and Author	Patient/ Center	Indication Aneurysm/ Dissection	Follow-Up	Technical Success, %	Type Ia Endoleak, N(%)	Stroke, N(%)	Operative Mortality, N(%)	RTAD, N(%)	Device Failure, N
**Najuta**									
Kawaguchi [15]	682(288) */1	474/208	5y	-	95.2% ^†^	26(3.8)	-	-	Migration 7.0%
Yokoi [33]	383/35	332/44	Perioperative	99.2	95.8% ^†^	7(1.8)	6(1.6)	3(0.8)	Collapse 1
Iwakoshi [34]	32/3	28/4	2.5 y	91	3(9.4)	1(3.1)	0	2(6.3)	LCA, LSA occlusion 1
Sato [35]	37/2	33/4	2.9 y	97.3	10(27.8)	6(16.7)	0	1	Migration 1
Iida [36]	20/1	20/0	19.9 m	95	2(10)	2(10)	0	0	0
Fukushima [24]	13/1	0/13	9.6 m	92.3	1(7.7)	0	0	0	0
Isernia [37]	76/21	61/6	7 m	97.4	1(1.3)	3(3.9)	1(1.3)	0	0
**TBE**									
Dake [38]	84/34	84/0	30 m	91.7	2(2.4)	3(3.6)	0	0	0
Dake [39]	31/6	31/0	25.2 m	100	1(3.4)	1(3.4)	0	0	SB for LSA occlusion 1

Type Ia endoleak and stroke occurred perioperative period. RTAD and device failure occurred during follow-up period. LCA: left common carotid artery. LSA: left subclavian artery. TBE: GORE TAG Thoracic Branch Endoprosthesis (W. L. Gore & Associates, Inc., Flagstaff, AZ, USA). SB: single side branch.

Yokoi et al. conducted a pilot study in 2010 and 2011, following the completion of the Najuta clinical trial. This study evaluated 383 patients with aortic arch diseases across 35 Japanese centers. All patients were considered to have serious risk factors for open surgical repair and were deemed ineligible for TEVAR with commercially available devices due to inadequate proximal landing zones. Of these patients, 332 had degenerative aneurysms, 44 had aortic dissections, 5 had traumatic transections of the aortic isthmus, and 2 had patent ductus arteriosus with congestive heart failure. Despite the average proximal sealing zone for the Najuta device being short at 14 mm, the procedures were completed with a mean operation time of 161 min, achieving a technical success rate of 99.2% and an initial success rate of 95.8%. The 30-day mortality rate was 1.6%, the incidence of cerebrovascular accidents was 1.8%, and the occurrence of ascending aortic dissection was 0.8%. Notably, all three patients with ascending aortic dissection had a history of previous aortic dissection and presented with a large-diameter (>40 mm) ascending aorta [33].

Iwakoshi et al. reported on the clinical outcomes of TEVAR using the Najuta fenestrated endograft in 32 patients with aortic arch diseases who were considered to have serious risk factors for open surgical repair. The technical success rate was 91%, with no perioperative deaths observed. Perioperative complications, including Stanford type A dissections, cerebral infarctions, and spinal cord ischemia, occurred in five patients. The rate of freedom from aneurysm-related death at 3 years was 97% [34].

Iida et al. reported the clinical outcomes of 20 patients treated with the Najuta system for arch or distal arch aneurysms between 2019 and 2023. The technical success rate was 95%, and two cases of type Ia endoleaks identified on postoperative CT scans disappeared spontaneously. Additionally, they noted two patients with stroke and two patients with spinal cord ischemia. Aortic events such as aneurysm enlargement, RTAD, and distal SINE were not observed during the follow-up period [36].

Fukushima et al. evaluated the outcomes of TEVAR for uncomplicated type B aortic dissection in 24 cases, divided into two groups: 13 cases with fenestrated Najuta stent grafts (F group) and 11 cases using debranching TEVAR with a standard stent graft (D group). The technical success rates were 92.3% in the F group and 100% in the D group, with no significant differences. However, the mean operation time and mean postoperative hospital stay were significantly shorter in the F group. The primary patency rate of the reconstructed branch vessel was 100% in both groups, with no aorta-related deaths or RTAD observed. Postoperatively, there was one case of a type Ia endoleak in the F group and one case of stroke in the D group, with no significant differences in the rate of major adverse events during the 14-month follow-up period [24].

Isernia et al. summarized the clinical outcomes of 76 cases of Najuta stent graft procedures performed at 21 vascular centers in Italy between 2018 and 2022. They reported a technical success rate of 97.4% and an initial clinical success rate of 94.7%, with no supra-aortic vessel occlusions or surgical conversions during the median 7-month follow-up period [37].

The Najuta has undergone more than seven years of PMS, with excellent clinical outcomes reported from over 600 cases performed at over 100 centers in Japan. Unfortunately, we are unable to provide detailed results for various reasons, including issues related to informed consent.

## 10. Comparison with Branched Stent Grafts

The Najuta fenestrated stent graft is not universally applicable to all types of aortic arch diseases. While it can often extend the proximal landing zone more than a standard stent graft, the IFUs specify that a favorable landing zone of 20 mm or more is required from the fenestration to the aneurysm. Kurimoto et al. performed TEVAR with the Najuta on 37 patients with aortic arch aneurysms, where the mean proximal landing zone length was 11.1 mm (range: 5–15 mm). They reported a type Ia endoleak in 12 patients (32.4%) at discharge. Subsequently, aneurysm enlargement was observed in six patients (16.2%), necessitating reintervention in four patients [40]. Since the Najuta primarily functions as a device to preserve the arch vessels using fenestrations, its range of indications is more limited compared to branched stent grafts, which can be placed in the ascending aorta with covered stents branching off to the arch vessels [41]. Therefore, in cases where the proximal landing zone is insufficient for the Najuta, a branched stent graft would be preferable.

Nevertheless, existing branched stent grafts cannot yet be considered ideal devices for aortic arch diseases [42]. Hauck et al.’s multicenter study (conducted across four tertiary aortic referral centers in Europe and Japan) compared 20 patients using branched stent grafts, with 34 patients using the Najuta fenestrated stent graft. They reported a perioperative mortality rate of 10% (two patients) for branched stent grafts and 0% for the Najuta, with strokes reported in 15% (three patients) and 2.9% (one patient), respectively. The study further found that 35% of patients suitable for a branched stent graft were also suitable for the Najuta device, highlighting the usefulness of the Najuta [43].

**Figure 13:**
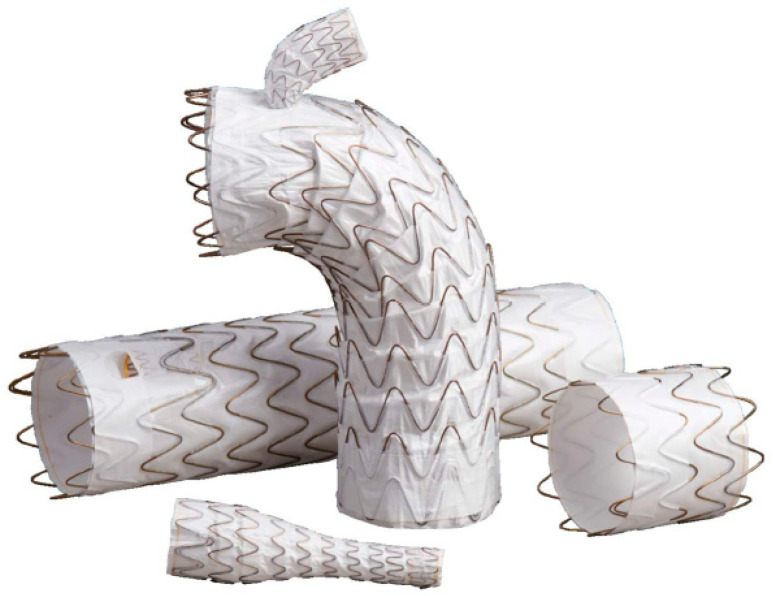
GORE TAG Thoracic Branch Endoprosthesis (TBE) (W. L. Gore & Associates, Inc., Flagstaff, AZ, USA) is the first single-branch stent graft modular device consisting of the aortic component, the side branch component, and an optional aortic extender composed of an expanded polytetrafluoroethylene (ePTFE) tube reinforced with ePTFE/fluorinated ethylene propylene film and supported by a self-expanding nitinol wire frame along its external surface (image courtesy of W. L. Gore & Associates G. K.).

Currently, the Thoracic Branch Endoprosthesis (TBE) (W. L. Gore & Associates, Inc., Flagstaff, AZ, USA) is the only branched stent graft approved for use in Japan (Figure 13). Because it has a single branch for the LSA, even if the main body is landed in the ascending aorta and a branch is placed in the BCA, a two-debranch bypass is required to reconstruct the LCA and LSA (Table 3). The approval of devices from other manufacturers with two branches is eagerly anticipated [38,39].

Ohki et al. have reported on the retrograde in situ branched stent grafting (RIBS) method for aortic arch aneurysms with insufficient proximal landing zones when using the Najuta. This technique involves creating branches by puncturing holes in a standard stent graft placed in the aortic arch with a needle introduced through the brachiocephalic artery (BCA) or LCA, followed by the retrograde insertion of covered stents. The study noted excellent clinical results with this method [44].

The enhancement of treatment outcomes depends on effectively leveraging the advantages of both branched and fenestrated stent graft designs while thoroughly evaluating the anatomical requirements. Further, long-term studies comparing Najuta with branched stent grafts are necessary.

## 11. Prospects of Endovascular Stent Graft Repair

As discussed, the semi-custom-made Najuta fenestrated stent graft offers potential applicability for aortic arch diseases when standard stent grafts have insufficient proximal landing zones. For example, in cases of arch aneurysms where a standard stent graft deployed in Zone 2 cannot secure the 2 cm proximal landing zone specified by the IFUs, the Najuta fenestrated stent graft can utilize the distance from the preserved LCA to the aneurysm as the proximal sealing zone. This enables a simplified TEVAR without the need for debranch bypass in many cases. Particularly in aneurysms located on the anterior or lesser curvature side of the aortic arch, the sealing zone from the LCA can be further extended. This should reduce the invasiveness for patients and lead to lower healthcare costs. We conclude that considering the Najuta as a treatment option before opting for hybrid TEVAR in complex aortic arch diseases offers significant advantages.

The limitations of the Najuta include the potential for endoleaks from the fenestration sites when treating aneurysm lesions located close to the LCA or BCA. In contrast, branched stent grafts allow for the placement of a covered stent branching from the main stent graft body into the arch vessels, with the main body extending to the ascending aorta. This makes branched stent grafts theoretically applicable to lesions located very close to the LCA or BCA. Additionally, while the semi-custom-made fenestrated stent graft may match the aortic anatomy in the early period, the long-term remodeling of the aortic arch could lead to misalignment between the fenestrations and the arch vessels, potentially causing vessel occlusion. Improvements that allow for the addition of branch-specific covered stents to the Najuta’s fenestrations are anticipated.

Several considerations and pitfalls are associated with the deployment method of the Najuta, which are not found with standard devices. The dual-wire tensioning technique used to straighten the curved sheath housing the pre-shaped Najuta for optimal sheath alignment carries the risk of dislodging atheroma from the arterial wall, particularly in the BCA, potentially causing embolization. Furthermore, the Z-stent inner skeleton has a relatively large individual piece length of 25 mm, which reduces its flexibility compared to standard stent grafts and may complicate reintervention efforts. Physicians operating with the Najuta are required to understand its characteristics and master its application and deployment techniques.

The Najuta received CE mark approval in 2017 and was initially implemented in Italy [36], followed by Germany and Austria. Plans exist for further expansion into Switzerland, the Netherlands, Poland, and the United Kingdom. In the approved countries, over 100 TEVAR procedures using the Najuta have already been performed, with more than 10 certified instructors emerging in these regions.

Since the development of stent grafts and their routine inclusion in treatment, approximately 20 years have passed. Both the techniques and devices are still in the process of development. Many clinical trials comparing long-term outcomes between conventional open surgery and endovascular stent graft repair note a higher incidence of reinterventions following endovascular procedures. As an endovascular repair method for aortic diseases, this technique is primarily aimed at being minimally invasive, leading to catheter-based interventions without excising the lesion, a treatment that cannot yet be considered radical. While comparable to conventional open surgery in terms of the freedom from aneurysm-related death, secondary interventions can lower patients’ quality of life, increase invasiveness and healthcare costs, and place higher demands on medical professionals.

However, as with all technologies, these medical devices are certain to advance rapidly. The development of branched stent graft devices capable of the flexible deployment of covered stents across various aortic vessels is anticipated to establish TEVAR as the primary treatment option for aortic arch and thoracoabdominal diseases. Additionally, the traditional system of attachment using the metallic stent’s radial force at the landing zones may evolve towards biochemical systems involving adhesion or fusion, diminishing stent graft-specific issues such as endoleaks and migrations and potentially eliminating secondary interventions altogether. We anticipate sustained device development in this therapy.

## Figures and Tables

**Table 1:** Characteristics of stent grafts approved in Japan.

Device Structure	GORE CTAG	COOK Alpha	COOK TxD	Medtronic Valiant	TERUMO RelayPro BS, NBS	SB-Kawasumi Najuta
Stent Material/Shape	Nitinol/ Spiral Z	Nitinol/Z	Stainless-steel/Z	Nitinol/Z	Nitinol/Z	Stainless-steel/Z
Graft Material	ePTFE	Woven polyester	Woven polyester	Woven polyester	Woven polyester	PTFE
Stent Mounting Side	Outside	Inside/ Outside	Inside/ Outside	Outside	Outside	Inside
Proximal Bare Stent	3–6.5 mm	15 mm	None	12 mm	BS: 15–21 mm NBS: Non	None

**Table 3:** Anatomic instructions for use of the GORE TBE and Najuta stent grafts.

**TBE**	**Proximal Aortic Landing Zone:** Aortic inner diameter 16–42 mm Proximal segment length (Length from distal edge of LSA to mid LCA ostium) 2.0–4.0 cm
	**LSA Landing Zone:** LSA inner diameter 6–18 mm Length 2.5–3.0 cm
	**Distal Landing Zone:** Aortic inner diameter 16–42 mm Outer curve length >2 cm proximal to celiac artery
**Najuta**	**Proximal Landing Zone:** Ascending aortic diameter 20–46 mm Proximal sealing zone (Between target vessels distal ostium and proximal edge of pathology) Length >20 mm, aortic inner diameter 20–40 mm
	**Distal Landing Zone:** Aortic inner diameter 20–40 mm Length >20 mm

## Data Availability

Data sharing is not applicable to this study as no new data were created.
